# Municipal resources to promote adult physical activity - a multilevel follow-up study

**DOI:** 10.1186/s12889-022-13617-8

**Published:** 2022-06-18

**Authors:** Virpi Kuvaja-Köllner, Eila Kankaanpää, Johanna Laine, Katja Borodulin, Tomi Mäki-Opas, Hannu Valtonen

**Affiliations:** 1 Department of Health and Social Management, University of Eastern Finland, POB 1627, FIN-70211 Kuopio, Finland; 2 Age Institute, Finland Jämsänkatu 2, 00520 Helsinki, Finland; 3 Finnish Institute for Health and Welfare, POB 30, FI-00271 Helsinki, Finland; 4 Department of Social Sciences, University of Eastern Finland, POB 1627, FIN-70211 Kuopio, Finland

**Keywords:** Physical activity, Population survey, Municipality, Resource allocation, Panel data, Multilevel model

## Abstract

**Background:**

In Finland, local authorities (municipalities) provide many services, including sports and physical activity facilities such as pedestrian and bicycle ways and lanes, parks, sports arenas and pools. This study aimed to determine whether local authorities can promote physical activity by allocating resources to physical activity facilities.

**Methods:**

The data on municipality expenditure on physical activity and sports, number of sports associations receiving subsidies from the municipality, kilometers of ways for pedestrians and bicycles and hectares of parks in 1999 and 2010 were gathered from national registers. These data were combined using unique municipal codes with individual survey data on leisure-time physical activity (*N* = 3193) and commuting physical activity (*N* = 1394). Panel data on physical activity originated from a national health survey, the Health 2000 study, conducted in 2000–2001 and 2011–2012. We used the data of persons who answered the physical activity questions twice and had the same place of residence in both years. In the data, the individuals are nested within municipalities, and multilevel analyses could therefore be applied. The data comprised a two-wave panel and the individuals were followed over 11 years.

**Results:**

The resources for physical activity varied between municipalities and years. Municipal expenditure for physical activity and total kilometers of pedestrian ways increased significantly during the 11 years, although a clear decrease was observed in individuals’ physical activity. In our models, individual characteristics including higher education level (OR 1.87) and better health status (OR 7.29) increased the odds of increasing physical activity. Female gender was associated with lower (OR 0.83) leisure-time physical activity. Living in rural areas (OR 0.37) decreased commuting physical activity, and age (OR 1.05) increased it. Women (OR 3.16) engaged in commuting physical activity more than men.

**Conclusions:**

Individual-level factors were more important for physical activity than local resources. A large part of the variation in physical activity occurs between individuals, which suggests that some factors not detected in this study explain a large part of the overall variation in physical activity.

**Supplementary Information:**

The online version contains supplementary material available at 10.1186/s12889-022-13617-8.

## Background

Physical inactivity is a contributory cause for a number of chronic conditions, such as cardiovascular diseases and type 2 diabetes [1–3]. Physical inactivity not only has a negative impact on health and quality of life, but also increases health care costs [4, 5]. For example, in Switzerland, physical inactivity was estimated to be responsible for 2% of disability-adjusted life years lost and 1.2% of total medical costs in the year 2013 [6]. The positive effects of physical activity (PA) on health have been broadly studied and are well known [7–9].

There is a growing belief that environmental and policy changes may be less expensive and more sustainable in changing the population’s health behaviour than actions at the individual level [10], but that these interventions will have even greater benefit if they are integrated with behavioural science [11]. The way that built environment and transport services are organised and planned may play a part in enabling PA [3, 12–14]. Policy-relevant PA research should provide information that helps decision makers focus on the issues that are most likely to increase PA at the population level [3, 15–19]. For example, when a living environment is more suitable for active transportation and sport and PA facilities are located nearby and are inexpensive and easy to access, individuals may be encouraged to make healthier behaviour choices [20]. On the other hand, there are still many open questions, such as whether active individuals seek out environments that support their PA interests, rather than built environments determining individual PA interests and participation [14]. There are only a few studies examining the correlation of governmental or local policies on PA [21], but their results are contradictory. One of the few studies, using Behavioral Risk Factor Surveillance System data from the United States of America, concluded that an increase in government spending on parks and recreation increased participation in team sports but reduced the time used for walking [22]. In Sweden, the availability of exercise facilities had a positive correlation with time spent on PA [23]. In contrast with this result, according to a study from the Netherlands, people with access to more green spaces walked and cycled less frequently and for fewer minutes than those with fewer green areas available [24]. Only commuting PA was higher among those with access to the greener living environment. Maas et al. [24] considered that a possible explanation for this result may be that in greener living environments, facilities such as shops are further away, and people are more likely to use a car to reach them.

Ruetten et al. [25] compared several European countries and suggested that countries with an infrastructure accommodating a broad variety of leisure-time physical activity (LTPA) and public policies for PA at the national level also have higher levels of LTPA. In a review by Pratt et al. [26], Finland was presented as a country with high investments in PA infrastructure. These investments might even have contributed to the increase in leisure-time sports in Finland between 1982 and 2012 [27]. Increases in urban density, mixed land use and access networks were associated with increased walking and cycling and decreased car use in one study conducted in Finland [28]. The evidence from 27 European countries showed that increased government expenditure on health promotion did not increase participation in sports. Rather, increased expenditure on education had a significant positive correlation with sports participation [29].

In Finland, due to the legislation, the governance structure is similar in all municipalities. The local authorities, i.e., municipalities, play a major role in providing many services, such as primary education, health and social services, and also PA facilities. Most of the decision-making related to PA settings, such as pedestrian and bicycle ways, parks, sports areas and public pools, as well as public support for PA societies and clubs, occurs at the local level. Municipalities have had self-government since the 1800s, and they levy taxes to fund the services provided, although some services require additional co-payments from the users. In addition to their own tax revenues, municipalities receive state subsidies for all public services, among others for PA, sports and outdoor recreation purposes [30, 31]. However, the role of these state subsidies is minor in financing the physical activities and facilities, covering only 3% of related costs [32]. Additionally, these subsidies are not earmarked. Municipalities can decide rather independently how they use the subsidy, and there are differences in what municipalities provide for their inhabitants. Thus, Finnish municipalities offer a unique opportunity to study the effect of local policies on PA.

The aim of this study was to determine whether allocation of resources to PA by local authorities has correlation with a population’s PA level. In other words, is it possible for a local authority to increase PA by allocating resources to various activities and an infrastructure that increases opportunities to be physically active during leisure time and while commuting? Our study combined follow-up data on both individuals and municipalities. This unique study design provided us with the opportunity to study the effect of local policies on PA.

## Methods

### Municipality resources for physical activity

The data on local authority resources for PA in 1999 and 2010 were gathered from the ‘Finances and activities of municipalities and joint municipal boards’ register maintained by Statistics Finland [33]. We used information on municipality expenditure on PA and sports and outdoor recreation; the number of sports associations receiving grants from the municipality; and the number of kilometres of pedestrian and bicycle ways and hectares of parks in the municipalities. The expenditure on PA/sport and outdoor recreation includes activities related to PA, sports and the outdoors, along with the provision of sports facilities, outdoor areas and routes. This includes, e.g., sports fields and halls, playgrounds, sports facilities, swimming beaches and other outdoor activities and the construction, maintenance and administration of these tasks. All monetary values were converted to euros and to the value level of the year 2020.

Finland is one of the most sparsely populated countries in Europe, and the population is highly concentrated in the southern and south-western parts of the country. In 2020, 72% of Finland’s overall population lived in urban areas and cities. The number of inhabitants and the density of population are associated with many factors relating to PA. In cities and urban areas, private provision of facilities is also common and in rural municipalities the longer distance to workplaces affects commuting PA. Therefore, we used the municipal classification, developed by Statistics Finland for describing the degree of urbanisation. This classification divides municipalities into three categories: (1) urban, (2) semi-urban and (3) rural municipalities [34]. In urban municipalities, at least 90% of the population lives in urban settlements or settings in which the population of the largest urban settlement is at least 15,000. In semi-urban municipalities at least 60% but less than 90% of the population lives in urban settlements and the population of the largest urban settlement is at least 4000 but less than 15,000. The rural municipality category means that either 1) less than 60% of the population lives in urban settlements and the population of the largest urban settlement is less than 15,000 or 2) at least 60% but less than 90% of the population lives in urban settlements and the population of the largest settlement is less than 4000 [34].

### Physical activity in population surveys Health 2000 and 2011

The data for PA originated from the population-based Health 2000 Study (*N* = 8028) [35] and its follow-up study in 2011 (*N* = 8135) [35, 36]. The data were pseudonymised, which means the processing of personal data in such a manner that the personal data can no longer be attributed to a specific person without the use of additional information and such additional information must be kept carefully separate from personal data. However, pseudonymised data can still be used to single individuals out and combine their data from different records. The Health 2000 and 2011 surveys were coordinated by the Finnish Institute for Health and Welfare (formerly National Institute for Health and Welfare). The original sampling frame comprised adults aged 30 years or older living in mainland Finland. These data were collected using a stratified two-stage cluster sampling design, and the data included all types of municipalities from the whole of Finland. The sampling frame was built around five university hospitals, each region containing about 1 million inhabitants. The units in the sample were either health centre districts (*N* = 80) or municipalities (*N* = 160). From every university hospital region, 16 health care centre districts were sampled as clusters. First, the 15 largest health centre districts were all selected in the sample, and the remaining 65 health centres were selected by systematic probability proportional to size sampling in each stratum. The full sampling procedure of the Health 2000 study and for Health 2011 Survey has been described in detail elsewhere [37, 38].

At the baseline, in 2000, the data included 161 municipalities, with 257 in 2011. The increase in the number of municipalities in the 2011 Health study data was due to internal migration. At the same time (especially 2005–2007), Finland has undergone several municipal reforms, which have resulted in municipal mergers. During this study period, the number of municipalities in Finland was reduced from 453 in 1999 to 336 in 2011. The number of municipal mergers related to Health 2000 and 2022 data were 32. In order to minimize the confounding effects of these mergers backgrounds, the panel data were presentedas if the municipal mergers had already taken place prior to 1999, by “allocating” the inhabitants in 1999 to the merged municipalities existing in 2011.

The survey was repeated in 2011. The data comprised a two-wave panel, and the same individuals were followed over a time span of 11 years. The outcome variables were leisure-time physical activity (LTPA) and commuting physical activity (CPA). The detailed questions are presented in the results section, Table 3; only a brief description of the questions is presented here. Intensity of LTPA was estimated with a multiple-choice question, in which subjects indicated the type of LTPA most often performed on a four-grade scale. In this question, we combined the options three and four because there were only very few observations in the highest PA level.

The original CPA question was a seven-grade scale multiple-choice question. For our analysis, the categories were reduced to five. First, we excluded those participants who reported “I do not work or I work at home”. Additionally, the two highest levels of CPA (1–2 hours per day and 2 hours or longer per day) were also merged due to the low numbers of observations. In addition, we used data on age, gender and education, and self-assessed health and municipality of residence.

### Data construction

The municipalities’ resource data from the year 1999 were combined with the population survey data of 2000 by using unique municipal codes. Similarly, the municipalities’ resource data from 2010 were combined with the population survey data of 2011.

**Fig. 1:**
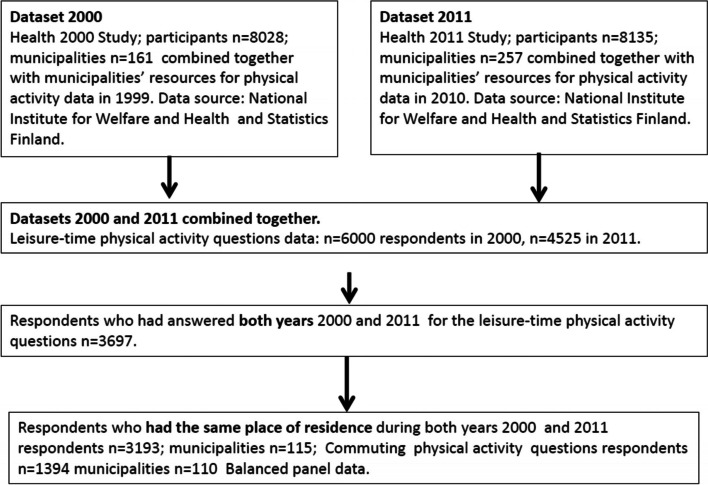
Formation of data

From the Health 2000 Study and its 8028 participants, 5903 also participated in the follow-up study in 2011 [37]. The reasons for the observed decline were the following: refusal to participate (16%), not contacted (10%), death (1%) and moving abroad (0.4%). For this study (Fig. 1), we included only individuals who had participated in the study both in 2000 and 2011 and had answered the LTPA questions (63%; *N* = 3697). Those participants who moved to another municipality during the follow-up period were excluded (about 14% of the of 3697). This criterion decreased the number of individuals to 3193, which represents 46% of the original sample. The number of municipalities with the LTPA question was 115. For the commuting physical activity (CPA) question, the number of respondents who answered twice, were still in working life, and did not work at home, was only 1394. The latter inclusion criteria decreased the number of municipalities in the CPA analysis to 110. The number of individuals in our data declined considerably from the original data. The major reason for this decline in the CPA question was that almost 60% of the participants either did not work anymore or worked at home, which is understandable since the average age of participants in this second survey, in 2011, was already 60 [35]. The number of participants included in the CPA question represents 17% of the original sample.

The inhabitants who moved to another municipality during this 11-year follow-up period were more educated, healthier and ca. Five years younger than those who did not change their place of residence during the follow-up period. The differences were significant. The gender and type of municipality of movers did not differ from each other.

### Statistical analysis

Multilevel, mixed-effects, ordinal logistic regression was applied due to the hierarchical structure of the data and the outcome measures being ordinal response variables. In a multilevel model, we could also include time-invariant variables such as gender and type of municipality, which would not be possible e.g., in a fixed-effects panel model [39].

For both PA variables, two models with stepwise inclusion of the explanatory variables were computed in addition to the ‘null model’. The null model includes no predictor variables. The ‘municipal resources model’ includes variables such as parks; pedestrian and bicycle ways; grants; municipal expenditure for PA; municipality type (urban, semi-urban or rural); and dummy variable for year. The third model, the ‘full model’, includes the municipal resources and the individual-level factors such as age, gender, education and health, as well as year.

The models were tested with the common multilevel model tests. First, the ratio of the variance in the intercept and its standard error was calculated for every model. If the between-municipalities ratio of variance is significantly different from zero, then this value should as a rule of thumb be greater than 2. After that, the intraclass correlation (ICC) was calculated. ICC expresses the proportion of the total variance at the municipal or individual level [40]. The ICC for ordinal outcomes can be calculated in the same way as for dichotomous variables. The level-one residuals are assumed to follow the standard logistic distribution that has a mean of 0 and a variance of π^2^/3 = 3.29. Then the ICC can be calculated with this equation: ICC = var1/(var1 + (π^2^/3)) [41].

Testing progressed with the log likelihood ratio tests, which can be used in at least two different ways. Firstly, after multilevel analysis, the log-likelihood test indicates whether the multilevel model is preferred over the single-level model or not. Secondly, it can be used to compare the nested models with each other. Finally, all the models were compared by using Akaike information criteria (AIC) and Bayesian information criteria (BIC) [41].

We tested whether there were differences in the resources for PA in the municipalities, in the characteristics of the study population, and in the PA levels of the population. We applied paired t-tests with equal variances for continuous variables and Pearson Chi2 ordinal multicategory variables. All statistical analyses were performed using Stata 15.

## Results

The kilometres of pedestrian and bicycle ways and municipal expenditure (€) for sports and outdoor recreation increased significantly, and the number of organisations receiving grants decreased significantly between the years 1999 and 2010 (Table 1). Of these 115 municipalities, 43 were urban, 25 semi-urban and 47 rural.

**Table 1:** Municipalities’ resources for physical activity

	1999 mean (SD)	2010 mean (SD)	Paired t-test
Number of municipalities	**115**	**115**	**115**
Park hectares/1000 inhabitants	2.83 (2.56)	2.88 (2.19)	0.836
Km of ways for pedestrians and bicycles/1000 inhabitants	1.49 (0.99)	1.94 (1.18)	0.000
Number of organizations receiving grants/1000 inhabitants	1.50 (0.83)	1.37 (0.84)	0.046
Municipal expenditure (€) for sports and outdoor recreation/inhabitant (2020 value)	73.78 (28.26)	98.06 (40.90)	0.000

Table 2 presents the characteristics of the study population in 2000 and 2011. The participants were naturally 11 years older in 2011, they were slightly more educated and there were also some minor changes in their self-assessed health. The share of population reporting moderate health status decreased and the shares below and above increased. Most of the participants were living in urban municipalities (62%). The rest of the participants were living in semi-urban (14%) and rural (24%) municipalities.

**Table 2:** The characteristics of the study population

Year	2000	2011	Significance test
**Number of participants**	3193	3193	*p*-value
**Proportion (%) of males**	45	45	1.00
**Mean age (SD)**	49.44 (11.83)	60.44 (11.83)	0.000
**What is your highest level of education completed after primary school (%)**	*N* = 3186	*N* = 3152	0.000
No vocational training or education at all	17	14	
Training or technical certificate for completed courses	17	17	
Vocational institution	32	31	
Technical college or special vocational qualification	20	19	
A degree of higher vocational qualification	4	7	
A higher university qualification, licentiate’s or doctor’s degree	9	11	
**Current health status (%)**	*N* = 3182	*N* = 3150	0.000
Poor	1	2	
Fairly poor	5	6	
Moderate	23	18	
Fairly good	32	35	
Good	39	39	
**Municipalities’ resources for physical activity among 3197 participants**			
Park hectares/1000 inhabitants	3.05	2.82	0.000
Km of ways for pedestrians and bicycles/1000 inhabitants	1.76	2.53	0.000
Number of organizations receiving grants/1000 inhabitants	1.27	1.16	0.000
Municipal expenditure (€) for sports and outdoor recreation/inhabitant (2020 value)	91.03	120.58	0.000

Table 3 presents the baseline and follow-up information about PA. There were significant statistical differences in LTPA levels between the years 2000 and 2011. The number of inactive individuals increased in LTPA. Due to the low number of respondents, we merged some response categories. In the CPA question, we excluded those participants who reported ‘I do not work or I work at home’. This decreased the number of observations from 2143 to 1394. In Table 3, we present the data with the original questions as phrased and the share of respondents in our recoded variables. The table with the original number of respondents is included in Additional file 1: Table S1.

**Table 3:** Physical activity in 2000 and in 2011

**Leisure-time physical activity (%)** ***N*** **= 3193**		
Year	2000	2011
In my leisure time I read, watch TV and do other activities in which I do not move much and which do not strain me physically.	23	30
In my leisure time, I walk, cycle and move in other ways at least 4 hours per week.	57	52
In my leisure time, I exercise at least 3 hours per week.	20	18
In my leisure time, I practise regularly several times per week for competition.		
	100%	100%
Pearson Chi^2^	0.000	
**Commuting physical activity variable (%)** ***N*** **= 1394**		
Year	2000	2011
I do not work, or I work at home	–	–
I use a motor vehicle for the entire trip	54	57
Less than 15 minutes per day	13	12
15–29 minutes per day	18	18
30–59 minutes per day	13	10
1–2 hours per day	3	3
2 hours or longer per day		
Pearson Chi^2^	0.181	

We present the results of three models (null, municipal resources and full) separately for LTPA (Table 4) and CPA (Table 5). Only the results of the null and full model are presented in the text. The main result for both LTPA and CPA is that individual-level factors were more important for PA than the municipalities’ resources for sports and outdoor facilities.

**Table 4:** Correlation of local authority resources and individual factors with leisure-time physical activity

Multilevel mixed-effects ordered logistic regression					
Leisure-time physical activity	Null model	Municipal resources model		Full model	
		OR	CI95	OR	CI95
**Year, reference 2000**		0.63**	0.54–0.73	0.59***	0.50–0.70
**Municipalities’ resources**					
Park hectares/1000 inhabitants		0.99	0.96–1.03	0.99	0.97–1.03
Km of ways for pedestrians and bicycles/1000 inhabitants		1.02	0.94–1.11	1.05	0.96–1.14
Number of organisations receiving grants/1000 inhabitants		0.94	0.83–1.06	0.95	0.85–1.06
€ used for sport and outdoor recreation/inhabitant		1.00	1.00–1.00	1.00	1.00–1.00
**Municipal type, reference urban**					
Semi-urban		0.81	0.63–1.04	0.88	0.70–1.11
Rural		0.78	0.57–1.05	0.93	0.69–1.24
**Age**				1.00	0.99–1.01
**Gender, reference male**				0.83*	0.72–0.97
**Education**, reference = no vocational education at all					
Training or technical certificate for courses completed				1.25	0.99–1.57
Vocational school				1.32*	1.57–4.85
A technical college or special vocational qualification				1.73***	1.35–2.22
A degree of higher vocational qualification				1.78***	1.27–2.50
A higher university qualification				1.87***	1.38–2.55
**Health**, reference poor					
Rather poor				0.99	0.54–1.82
Moderate				2.76***	1.57–4.85
Rather good				4.50***	2.57–7.90
Good				7.29***	4.14–12.84
Municipal-level variance (Standard Error)	0.051(0.030)	0.022 (0.024)		0.010 (0.018)	
Individual-level variance (Standard Error)	2.486 (0.193)	2.585 (0.199)		2.239 (0.186)	
Municipal-level intraclass correlation	0.015	0.007		0.003	
Individual-level intraclass correlation	0.430	0.440		0.405	
Number of observations	6394	6394		6394	
Number of groups (individuals)	3197	3197		3197	
Observation per group: minimum/average/maximum	2/2/2	2/2/2		1/2/2	
Number of groups (municipalities)	115	115		115	
Observation per group (per municipality): min/average/max	2/55.6/650	2/55.6/650		2/55.1/641	
Wald chi2/Prob>chi2		71.24***		346.66***	
Linear regression test vs. ologit regression	455.65***	462.50***		362.27***	

^*^p ≤ 0.05

^**^p ≤ 0.01

^***^p ≤ 0.001

**Table 5:** Correlation of local authority resources and individual factors with commuting physical activity

Multilevel mixed-effects ordered logistic regression					
Commuting physical activity	Null model	Municipal resources model		Full model	
		OR	CI95	OR	CI95
**Year, reference 2000**		0.66**	0.50–0.86	0.36***	0.25–0.51
**Municipalities’ resources**					
Park hectares/1000 inhabitants		1.01	0.95–1.07	1.01	0.96–1.07
Km of ways for pedestrians and bicycles/1000 inhabitants		1.07	0.93–1.22	1.07	0.93–1.23
NR of organisation receiving grants/1000 inhabitants		0.86	0.68–1.09	0.85	0.67–1.07
€ used for sport and outdoor recreation/inhabitant		1.00	1.00–1.00	1.00	1.00–1.01
**Municipal type, reference urban**					
Semi-urban		0.64	0.39–1.05	0.71	0.44–1.15
Rural		0.36***	0.20–0.66	0.38***	0.21–0.68
**Age**				1.06***	1.04–1.08
**Gender, reference male**				3.16***	2.36–4.16
**Education**, reference no vocational education at all					
Training or technical certificate for courses completed				1.08	0.63–1.85
Vocational school				0.86	0.52–1.41
A technical college or special vocational qualification				0.67	0.40–1.12
A degree of higher vocational qualification				0.69	0.37–1.27
A higher university qualification				1.40	0.79–2.46
**Health**, reference poor					
Rather poor				0.69	0.09–5.40
Moderate				0.91	0.13–6.42
Rather good				1.25	0.18–8.73
Good				1.21	0.17–8.43
Municipal-level variance (Standard Error)	0.505(0.168)	0.153 (0.105)		0.164 (0.106)	
Individual-level variance (Standard Error)	4.070 (0.433)	4.076 (0.433)		3.579 (0.398)	
Municipal-level intraclass correlation	0.133	0.044		0.048	
Individual-level intraclass correlation	0.552	0.553		0.521	
Number of observations	2788	2788		2774	
Number of groups (individuals)	1394	1394		1394	
Observation per group: minimum/average/maximum	2/2/2	2/2/2		1/2/2	
Number of groups (municipalities)	110	110		110	
Observation per group (per municipality): min/average/max	2/25.3/304	2/25.3/304		2/25.2/303	
Wald chi2/Prob>chi2		44.78***		144.01***	
Linear regression test vs. ologit regression	413.76***	336.47***		282.61***	

^*^p ≤ 0.05

^**^p ≤ 0.01

^***^p ≤ 0.001

The results of the null model for LTPA revealed that there was significant between-group variance of 0.051, the intercept variance across all municipalities. However, the ICC indicated that only 1.5% of the overall variance was accounted for by municipalities. The log-likelihood test suggested that the multilevel model was preferrable over the single-level model. Furthermore, the AIC and BIC tests (Additional file 1: Table S2) showed that the models with more variables and levels would be preferred. In the following results text, the OR value indicates the odds of being above a particular (next) PA level.

For LTPA, in the full model, a higher education level (OR 1.87) and better self-assessed health (highest level of health: OR 7.29) increased the likelihood for being above a particular level of PA (Table 4). Women engaged in less LTPA than men (OR 0.83). The year variable was also significant; in 2011, the LTPA was lower than in 2000.

In the full model, the municipal-level ICC was less than 2%, which indicates that less than 2% of total variance is accounted for by municipalities. On the individual level, the ICC indicated that 40–44% of overall variance is accounted for by individuals.

For CPA, the results of the null model revealed significant between-group variance of 0.505, the intercept variance across all municipalities. The log-likelihood test indicated that the multilevel model was preferrable over the single-level model. The ICC indicated that 13% of the overall variance was accounted for by municipalities.

Municipalities’ resources did not have any correlation with CPA levels. However, the type of municipality, in addition to individual factors, proved to be significant. In the full model, ageing increased the likelihood of CPA (OR 1.06; Table 5). In the rural municipalities, people reported less CPA than in urban municipalities. Women practiced more CPA (OR 3.16) than men. Those individuals who lived in rural areas practiced less CPA (OR 0.38) than those living in urban areas. Once again, the change from the reference year 2000 to 2011 decreased the CPA (OR 0.36). The ICC varied at the municipal level between 4 and 13%. At the individual level it varied between 52 and 55%.

## Discussion

The resources for PA varied between municipalities, but these differences did not explain the variation in individuals’ PA. There was a ‘municipal-level effect’ for CPA, which however was not related to municipalities’ resources but to the environment in the rural municipalities. The resources provided by local authorities had no correlation with the PA of individuals. Individual-level factors and type of municipality were much more important in explaining PA levels.

Our unique study design makes comparison with earlier studies challenging. The study exploring the effect of government spending on sports participation in 27 European countries yielded rather similar results. Government spending on health promotion did not increase PA. By contrast, spending on education had a significant positive correlation with sports participation [29]. Humphreys and Ruseski [22] concluded that increased government spending on parks and recreation increased participation in team sports, but decreased the time used for walking. In Finland, parks may not be very important for PA due to the plenitude of forests available for recreational purposes. In Stockholm, study results have indicated that the availability of exercise facilities (at least four exercise facilities within a 1000-m road network) has a positive correlation with time spent on PA [23]. In this study, we did not have the opportunity to examine the distances to the nearest PA facilities. However, some earlier studies in Finland have demonstrated that the environment may play an important role in LTPA and CPA [42, 43]. The aim of our study was to assess the correlation of public locally provided resources with PA. Many private providers also exist, which can function as substitutes for public facilities. However, we could not take private resources into account in this work. Pratt’s comment [26] that Finland has already invested considerably in PA infrastructure might be relevant. Perhaps the basic infrastructure is already good enough for those who enjoy PA, but for those who do not get any enjoyment from PA, the increase in resources and facilities alone will not change their PA behaviour. One of the biggest and most remarkable differences between rural and urban areas in Finland is the population number and density, and thus distances. In the rural area, distances to the workplace, but also to hobbies, are often much longer than in urban or semi-urban areas. Therefore, the results for CPA, indicating that people in the rural areas are practicing less CPA than those in urban areas, sounds logical. On the other hand, there were no differences between these areas in the LTPA. Either there are sufficient facilities in the rural area, or people in rural areas practice different kinds of physical activities than people in urban areas. Additionally, the proximity of nature and of the countryside provides various and different options than those in cities for people to be physically active in ways which are not provided by municipalities or private providers. Most (62%) of the participants of this study were living in the urban municipalities. This figure is very close to the share of urban housing in Finland, which makes it possible to generalize the results of the study.

As the results of this study indicate, most of the variation occurs between individuals (individual heterogeneity), which suggests that some factors not detected in this study explain a large part of the overall variation in PA. Allocation of resources to the PA facilities is needed, as without a facility there would be zero participants. Importantly, the decision-makers should know better whether the demand meets individuals’ needs and motivation towards a physically active lifestyle, and allocate local resources accordingly. The supply of PA facilities by local authorities is needed especially from the point of view of equity. But as we can see, local supply alone is not enough Ultimately, it is the question of individual preferences, motivations and demand for PA which should be studied more carefully. It is also important to consider which factors affect in which direction – do active individuals seek out environments that support their PA interests, or does the built environment determine individual PA interests and participation [13].

The strength of this study was the multilevel data setting and the possibility to use both individual-level and municipality-level follow-up data. The data on PA are based on a national, representative and large survey, albeit only for persons over 30 years of age in 2000. Although our data did not include all Finnish municipalities, it did include a large and representative sample of the municipalities. Despite the fact that we did not have detailed data on public sports facilities, this problem is remedied at least partially by the fact that municipal expenditures also include the costs of running these facilities. Further continuation and future research into this subject could focus on municipalities where the PA level was higher (and lower) than in others, and the topic could be explored by conducting, for example, field research and interviews in these municipalities. Information about active and inactive municipalities is available in the data used in this study. In addition, in forthcoming studies, it will be possible to use the information about all public and private sports facilities, routes and recreational areas and facilities, as the information has been made available from the year 2010 onwards. Combining a geographic information system and detailed data on locations to study geographical access to facilities and built environments would be very useful in further studies. More detailed research is also needed to determine the motivational backgrounds for PA. This might help us to understand how it is possible to promote PA, especially among non-active individuals.

The current findings should be interpreted with a degree of caution due some limitations of this study. Firstly, we did not have detailed data on the number of public sport facilities. On the other hand, this problem was remedied at least partially by the fact that municipal expenditures also include the costs of running these facilities. Secondly, we could not take private PA facilities and resources into account. It is obvious that many private providers exist, and they function as substitutes for public facilities. Thirdly, the distances to both public and private PA facilities plays a role in the possibility to use these services, which was not taken into account in this study. Fourthly, Finland has undergone several municipal reforms, which have yielded municipal mergers. This merging of the municipalities caused methodological challenges in processing the statistics. The decision to keep solely those individuals in the analysis who had the same place of residence during both survey times reduced the number of observations in this study. This might have caused bias to the results. Fifthly, during the 2011 Health survey, the mean age of the participants was already 60, which had a major impact on the number of participants in the CPA questions. For this reason, the results for CPA are only indicative. Although the original sample was representative for the Finnish adult population, our follow-up data suffers from selection problems, not so much for the municipalities themselves but more for the individuals in our data, which could not be remedied in our study.

Despite these limitations, this was a rich and representative data to study the use of municipal level resources for physical activity, and their correlation with PA. The data for the LTPA question can still be seen as a quite representative sample of Finnish municipalities and their inhabitants over 30 years of age, although it was less representative for CPA. Although the data was of high quality, we agree that some factors may still have remained unobserved.

Hopefully, this study will encourage researchers in other countries to exploit registers of this type and individual level data, in order to conduct similar studies. The aim could be to extend from policy recommendations and their association with physical activity levels to register-based data, such as used here for municipality resource data.

## Conclusions

Differences in PA are primarily associated with individual characteristics, such as higher education level, better health status, gender, age and municipality type: people living in urban areas engage in more CPA than people in rural areas.

The resources for PA varied between municipalities, but these differences did not explain the variation in individuals’ PA. This leads to us to the conclusion that it is important is to determine the most effective ways to increase PA among inactive inhabitants and then implement these effective and cost-effective activities and allocate the resources accordingly. Integration of the economics view to PA research could provide information on how to allocate public resources in order to increase PA at the population level. The use and implementation of effective and cost-effective interventions to promote PA are essential.

The determinants and mechanisms behind exercise and PA are complex. There is obviously a need for local resources-based research with better data (spatial data, private supply). There is a considerable amount of research related to individual level PA and its correlation with e.g., socioeconomic factors, but the research lacks this dimension of the supply environment and resources offered at the local level. Furthermore, in this study, a large part of the variation occurred between individuals, and the differences between PA were probably explained by some other factors not measured in this study. In the future, it would be important to try to understand the diversity of PA promotion and to enrich PA research with behavioural economics, with questions such as the individual’s demand for PA and PA preferences.

## Supplementary Information

**Additional file 1: Table S1.** The original and recoded physical activity answers of participants. **Table S2.** The results of AIC and BIC statistics.

## Data Availability

Health 2000/2011 data is available for research purposes from the Finnish Institute for Health and Welfare after the research proposals have been accepted. The data for Finances and activities of municipalities and joint municipal boards for years 1999 and 2010 were publicly available from Statistics Finland until 2016, but anymore. https://www.stat.fi/tup/alue/kuntien-raportoimat-tiedot_en.html At the moment the data is available from year 2015. https://pxnet2.stat.fi/PXWeb/pxweb/en/StatFin/StatFin__jul__kta/statfin_kta_pxt_12mk.px/

## Abbreviations

PA: Physical activity
LTPA: Leisure-time physical activity
CPA: Commuting physical activity
ICC: Intraclass correlation
AIC: Akaike information criteria
BIC: Bayesian information criteria
