# Supplementary material for: Municipal resources to promote adult physical activity - a multilevel follow-up study
Source: BMC Public Health. 2022 Jun 18;22:1213. doi: 10.1186/s12889-022-13617-8 (PMC9206276; doi:10.1186/s12889-022-13617-8)
Supplement: Supplementary file 1 — Additional file 1: Table S1. The original and recoded physical activity answers of participants. Table S2. The results of AIC and BIC statistics. [file 12889_2022_13617_MOESM1_ESM.docx]

Additional file 1.

Table S1. The original and recoded physical activity answers of participants

| Leisure time physical activity | | | | |
| --- | --- | --- | --- | --- |
|  | Original variable (%) | | New recoded 3-class variable | |
|  | N=3 193 | | N=3 193 | |
| Year | 2000 | 2011 | 2000 | 2011 |
| In my leisure time I read, watch TV and do other activities in which I do not move much and which do not strain me physically. | 23 | 30 | 23 | 30 |
| In my leisure time, I walk, cycle and move in other ways at least 4 hours per week. | 57 | 52 | 57 | 52 |
| In my leisure time, I exercise at least 3 hours per week. | 19 | 17 | 20 | 18 |
| In my leisure time, I practise regularly several times per week for competition. | 1 | 1 |  |  |
|  | 100 % | 100 % | 100 % | 100 % |
| Pearson Chi^2^ | 0.000 | | 0.000 | |
| Commuting physical activity | | | | |
|  | Original variable (%) | | New recoded 5-class variable (%) | |
| Number of individuals who have responded twice to the survey | N=2 142 | | N=1 394 | |
| Year | 2000 | 2011 | 2000 | 2011 |
| I do not work or I work at home | 34 | 34 | - | - |
| I use a motor vehicle for the entire trip | 36 | 38 | 54 | 57 |
| Less than 15 minutes per day | 8 | 8 | 13 | 12 |
| 15-29 minutes per day | 12 | 12 | 18 | 18 |
| 30-59 minutes per day | 8 | 7 | 13 | 10 |
| 1-2 hours per day | 2 | 1 | 3 | 3 |
| 2 hours or longer per day | <1 | <1 |  |  |
| Pearson Chi^2^ | 0.019 | | 0.181 | |

Table S2. The results of AIC and BIC statistics

| **Leisure time PA model** | Obs | df | AIC | BIC |
| --- | --- | --- | --- | --- |
| Null model | 6 394 | 4 | 12 317.25 | 12 344.31 |
| Municipal resources model | 6 394 | 11 | 12 259.69 | 12 334.09 |
| **Full model** | **6 394** | **22** | **11 861.92** | **12 010.53** |
|  |  |  |  |  |
| **Commuting PA** |  |  |  |  |
| Null model | 2 788 | 6 | 6 501.43 | 6 537.03 |
| Municipal resources model | 2 788 | 13 | 6 476.58 | 6 553.71 |
| **Full model** | **2 788** | **24** | **6 351.53** | **6 493.80** |
|  |  |  |  |  |
